# Adipocytic Glutamine Synthetase Upregulation *via* Altered Histone Methylation Promotes 5FU Chemoresistance in Peritoneal Carcinomatosis of Colorectal Cancer

**DOI:** 10.3389/fonc.2021.748730

**Published:** 2021-10-12

**Authors:** Xuan Zhang, Qing Li, Aibei Du, Yifei Li, Qing Shi, Yanrong Chen, Yang Zhao, Bin Wang, Feng Pan

**Affiliations:** ^1^ Department of Oncology, Southwest Hospital, Army Medical University (Third Military Medical University), Chongqing, China; ^2^ Department of Gastroenterology, Daping Hospital, Army Medical University (Third Military Medical University), Chongqing, China; ^3^ Department of Science and Education, The People’s Hospital of Tongliang District, Chongqing, China; ^4^ Department of Hematology, The Second Affiliated Hospital of Chongqing Medical University, Chongqing, China; ^5^ Department of Respiratory Medicine, The People’s Hospital of Tongliang District, Chongqing, China

**Keywords:** peritoneal carcinomatosis, colorectal cancer, chemoresistance, glutamine synthetase, histone methylation

## Abstract

The development of resistance to 5-fluorouracil (5FU) chemotherapy is a major handicap for sustained effective treatment in peritoneal carcinomatosis (PC) of colorectal cancer (CRC). Metabolic reprogramming of adipocytes, a component of the tumor microenvironment and the main composition of peritoneum, plays a significant role in drug resistance of PC, with the mechanisms being not fully understood. By performing metabolomics analysis, we identified glutamine (Gln), an important amino acid, inducing resistance to 5FU-triggered tumor suppression of CRC-PC through activating mTOR pathway. Noteworthily, genetic overexpression of glutamine synthetase (GS) in adipocytes increased chemoresistance to 5FU *in vitro* and *in vivo* while this effect was reversed by pharmacological blockage of GS. Next, we showed that methionine metabolism were enhanced in amino acid omitted from CRC-PC of GS transgenic (Tg^GS^) mice, increasing intracellular levels of S-carboxymethy-L-cys. Moreover, loss of dimethylation at lysine 4 of histone H3 (H3k4me2) was found in adipocytes *in vitro*, which may lead to increased expression of GS. Furthermore, biochemical inhibition of lysine specific demethylase 1 (LSD1) restored H3k4me2, thereby reducing GS-induced chemoresistance to 5FU. Our findings indicate that GS upregulation-induced excessive of Gln in adipocytes *via* altered histone methylation is potential mediator of resistance to 5FU chemotherapy in patients with CRC-PC.

## Introduction

Colorectal cancer (CRC) is the third most commonly diagnosed cancers and the fourth leading cause of cancer-associated mortality worldwide (1, 2). Metastasis is the main cause of poor outcome in CRC patients. Among all the metastasis access, the peritoneum is the second most common site for CRC metastasis, and 4.8% of CRC patients exhibit evidence of synchronous peritoneal carcinomatosis (PC) while 19% show characteristic of metachronous PC (3, 4). PC is a well-known indicator of poor prognosis of CRC, and the median survival time of patients with PC is only 6–11 months (4, 5).

5-fluorouracil (5FU), a thymidylate synthase inhibitor, is often used during early intraperitoneal chemotherapy. 5FU-based chemotherapy is a standard treatment for patients with metastatic CRC (6). 5FU acts during the S-phase of the cell cycle and blocks purine synthesis by inhibiting thymidylate synthetase activity, thereby reducing DNA replication and repair, leading to suppression of tumor cell growth (7, 8). However, resistance to 5FU treatment in patients with CRC remains common, promoting tumor recurrence and metastasis (9), with the mechanisms being not fully known. Therefore, it is crucial to get a better understanding of the underlying mechanisms of resistance to 5FU chemotherapy for the management of patients with CRC- PC.

Tumor microenvironment (TME), a heterogeneous ecosystem contributing to tumor progression, consists of infiltrating immune cells, mesenchymal support cells, and matrix components. As the important component of TME, adipocytes play essential roles in facilitating tumor growth and mediating drug resistance (10). However, it is still unclear whether adipocytes, the main composition of peritoneum, mediate resistance to 5FU chemotherapy in CRC-PC. Besides, as abnormal adipocyte metabolism in dysfunctional adipose tissue induces the development of cancers and drug resistance (11), and 5FU chemoresistance may result from altered regulation of nucleotide metabolism, amino acid metabolism, and oxygen metabolism (12), we hypothesized that adipocyte-derived cytokines or metabolites might contribute to chemoresistance in CRC-PC.

Glutamine (Gln), one of the adipocyte-derived metabolites, has been found to mediate chemoresistance by several mechanisms (13). Drug resistance caused by increased intracellular glutamine content is directly associated with the dynamic change of glutamine transporters (14). Glutamine has been also shown to promote chemoresistance *via* the mammalian target of rapamycin (mTOR) activation (15). Glutamine synthetase (GS), an ATP-dependent metalloenzyme, combines ammonium and glutamate into Gln and is associated with chemoresistance (16). Although common mechanisms leading to 5FU resistance have been well-documented in the literature, the role of Gln and GS in 5FU resistance needs to be clarified.

mTOR, a specificity protein kinase phosphorylating serine/theonine, plays substantial roles in cell proliferation, survival, autophagy, and metabolism (17). Dysregulation and activating mutations of mTOR have been reported in various types of human cancers, and hence mTOR inhibitors have been approved for the treatment of malignancies (18). mTOR signaling also plays important roles in drug resistance of CRC (19). As a drug-resistance-related protein, mTOR mediates 5FU drug resistance (20).

Histones are basic chromosomal proteins that play essential structural and functional roles in gene regulation and epigenetic silencing (21). Histone proteins can be reversibly modified by methylation, acetylation, ubiquitination, and phosphorylation. These modifications of histone structures have been shown to play crucial roles in cancer initiation and progression with different mechanisms (22). Histone methylation can be capable of altering chromatin structure and regulating gene expression. Additionally, histone methyltransferase activity has been found to be involved in 5FU chemoresistance in CRC (9, 23). However, at this time, it remains unclear whether altered histone methyltransferase activity might affect adipocyte metabolism and mediate 5FU resistance in CRC-PC. In order to improve the therapeutic efficacy of 5FU and elucidate the mechanism underlying the development of 5FU resistance in patients with CRC, we firstly identified glutamine (Gln) as an important amino acid to induce resistance to 5FU-triggered tumor suppression of CRC-PC *via* activating mTOR pathway using metabolomics analysis. We then examined whether altered genetic expression of glutamine synthetase (GS) in adipocytes can affect chemoresistance to 5FU *in vitro* and *in vivo*. Next, we explored methionine metabolism in amino acid omitted from CRC-PC of GS transgenic (Tg^GS^) mice and the underlying mechanisms of 5FU resistance through regulation of histone H3 lysine 4 dimethylation (H3k4me2) in adipocytes *in vitro*.

## Materials and Methods

### Cell Culture

The mouse CRC cell lines CT26, MC38, mouse melanoma cell line B16, human CRC cell lines SW-480 and HCT-116 were purchased from American Type Culture Collection (ATCC, Rockville, MD, USA). Those cells have been authenticated and checked for mycoplasma by JENNIO Biological Technology (Guangzhou, China). The cell lines were maintained in continuous exponential growth by twice weekly passage in Dulbecco modified Eagle’s medium (DMEM, Life Technologies, Inc., Gaithersburg, MD, USA) supplemented with 10% fetal bovine serum and cultured at 37°C with 5% CO2. Each cell line was split regularly before attaining 70–80% confluence.

The “cocktail method” was used to induce differentiation based on optimization of a method previously described (24, 25). Briefly, the 3T3-L1 cells (2 × 10^5^ cells/ml) were grown to confluence in DMEM with 10% FBS in T75 cell culture flask (day −2). On day 0, the media were changed to DMEM with supplements plus 10% fetal bovine serum, 10 μg/ml insulin (Sigma-Aldrich; Merck KGaA, Darmstadt, Germany), l μmol/L dexamethasone (Sigma-Aldrich; Merck KGaA, Darmstadt, Germany), and 0.5 mM isobutylmethylxanthine (IBMX, Sigma, St. Louis, MO, USA). On day +2, dexamethasone and IBMX were removed from the media, and on day +4 insulin was removed. Media changes were performed every 2 days until use. The CM from adipocytes (#3T3-L1-CM) were harvested between days +10 and +14.

### Mouse Experiments

All the animal experiments were approved by the Laboratory Animal Welfare and Ethics Committee of Army Medical University (AMUWEC20181835) and performed in accordance with the *Guide for the care and use of laboratory animals* published by the US National Institutes of Health (publication no.85-23, revised 1996). Four-to-six-week-old female BALB/c mice (body weight: 18–20 g) were purchased from the Institute of Experimental Animal of Army Medical University (Chongqing, China). The adipocyte-specific transgenic GS-expressing C57BL/6 mice (Cyagen Biosciences, China) were generated by inserting a DNA pRP(Exp)-Promoter_5411bp (Adiponectin)>mGlul[ORF031394]. Primers for PCR genotyping of the GS forward: GACATGATGCAGGTCCTGATTGG; reverse: GGGTCTTGCAGCGCAGTCCTT. Transgenic mice yield a PCR product of 248 bp. Mice were maintained under specific pathogen-free conditions and had *ad libitum* access to food and water. CT26 cells (1.0 × 10^6^/100 μl PBS) were intraperitoneally injected into female BALB/c mice, or MC38 cells (1.0 × 10^6^/100 μl PBS) were intraperitoneally injected into GS-expressing C57BL/6 mice to establish a peritoneal metastasis model according to Abdelkader Taibi et al. (26) and Liping Yang et al. (27). CT26 cells (1.0 × 10^6^/100 μl PBS) were injected subcutaneously into female BALB/c mice to establish a subcutaneous xenograft model. CT26 cells (1.0 × 10^6^/100 μl PBS) were also injected into the colon to establish a *in situ* model of CRC. 5FU was administrated intraperitoneally once a day (50 mg/kg). Different kinds of conditional medium derived from adipocytes were administered around the basement of the tumors (s.c., 100 μl/2d) at the start of 5FU treatment when the tumor diameter was around 0.5 cm. Furthermore, GS antagonist L2A (#A7275, Sigma, USA), LSD1 inhibitor GSK-LSD1 (#SML1072, Sigma, USA), and mTOR inhibitor Rapamycin (#SML2282, Sigma, USA) were also tested *in vivo* for their ability to interfere with the induction of resistance. L2A (1 mg/kg) was administered intraperitoneally every other day for three times. GSK-LSD1 (0.5 mg/kg) was administered intraperitoneally every day for 7 days. Rapamycin (10 mg/kg) was administered intraperitoneally every day for 7 days. Tumor growth was monitored every day by measuring diameters using Vernier caliper. After 18 days after injection, mice were sacrificed followed by dissection and assessment of PC. Then the tumors were calculated in weight.

### Metabolomics

Fat tissues were collected from the peritoneum of the peritoneal metastasis model (n = 3) and the *in situ* CRC model (n = 3). Fat tissues from peritoneum of WT mice (n = 6) and Tg^GS^ mice (n = 6) were also collected. Metabolomics were performed in fat tissues using gas chromatography/time-of-flight mass spectrometry (GC-TOF/MS) measurement (Agilent 7890B-LECO Pegasus HT/BT). The Chroma TOF4.3X software (LECO) and LECO-Fiehn Rtx5 database were used for raw peaks extraction, data baselines filtering, and calibration. The resulted three-dimensional data involving the peak number, sample name, and normalized peak area were fed to SIMCA14 software package (Umetrics, Umea, Sweden) for principal component analysis (PCA) and orthogonal projections to latent structures-discriminate analysis (OPLS-DA). The identified differential metabolites (fold change >2 or <0.5, P < 0.05) were used to perform heatmap analysis. TBtools software was used to perform heatmaps of differential metabolites (28).

### Cell Counting Kit-8 Assay

The cell viability in 5FU treatment was measured by performing CCK8 assay. The CRC cells including CT26, MC38, SW-480, and HCT-116 cells and melanoma cells B16 were trypsinized and seeded at 1.0 × 10^4^ cells/well in 96-well plates, respectively. After overnight incubation, the medium was removed and replaced with different kinds of conditional medium derived from adipocytes and 5FU (10 μmol/l) for chemotherapy. At different time points, 10 μl of CCK8 solution (Dojindo Laboratories, Japan) in PBS was added into each well. Plates were incubated at 37°C for another 1 h. The optical density for each well was measured using a microculture plate reader (BioTek, USA) at absorbances of 450 nm.

### Cell Cycle Arrest Assay

Cell cycle arrest assay was performed using a cell cycle assay kit (C1052, Beyotime, China).

Briefly, the CT-26 cells were digested to single cells and washed twice with cold PBS. Then, the cells were fixed with cold ethanol (75%) for 12 h and washed with cold PBS. Finally, the cells were stained with propidium iodide for 30 min before flow cytometry analysis.

### Annexin V Apoptosis Assay

Cell apoptosis assay was performed using Annexin V staining (AO2001-02A-H, Sungene Biotech, China). CT26 cells were trypsinized and seeded at 2.0 × 10^5^ cells/well in six-well plates. After overnight incubation, the medium was removed and replaced with different kinds of conditional medium derived from adipocytes and 5FU (10 μmol/l) for chemotherapy. Each well was added with annexin V-FITC and was incubated at 37°C for 15 min in the dark. The annexin V-FITC binding was detected by flow cytometry (FACSAria, BD Bioscience) using FITC signal detector (FL1) and PI staining by the phycoerythrin emission signal detector (FL2).

### Knockdown of Glutamine Synthetase by RNA Interference

The specific siRNA targeted mouse GS (si-GS: 5’- CCACCTCAGCAAGTTCCCACTTGAA- 3’) and a scrambled control siRNA that had no sequence homology to any known genes (5’ - CCAGACTGAACCCTTTCACTCCGAA - 3’) were designed and synthesized by Qiagen (Shanghai, China).

### Knockdown of mTORC1 by shRNA

Plasmid pGCsi-U6-Neo-GFP-shRNA Expression Vector (GeneChem) was used. We designed two pairs of complementary oligonucleotide sequences (shRNA#1 and shRNA#2) according to the cDNA sequences of mTORC1 (GenBank Accession Number: NM_028022.2). The scrambled control plasmid was a circular plasmid encoding a shRNA which had the sequence not present in the mouse, human, or rat genome databases. The sequences are shown below:

shRNA#1:
5’- CACCGCAGTCGGTGCAAGTTCTTCACGAATGAAGAACTTGCACCGACTGC -3’
5’- AAAAGCAGTCGGTGCAAGTTCTTCATTCGTGAAGAACTTGCACCGACTGC -3’
shRNA#2:
5’- CACCGGTACATCTCCATTGTCATGGCGAACCATGACAATGGAGATGTACC -3’
5’- AAAAGGTACATCTCCATTGTCATGGTTCGCCATGACAATGGAGATGTACC -3’

### Real-Time PCR

Total RNAs were isolated using a peqGold Total RNA Kit including DNase digestion

(Peqlab, Erlangen, Germany). RNAs were transcribed into cDNAs using Omniscript

(Qiagen, Hilden, Germany). qPCR was performed using the 7900 HT Fast Real-Time

PCR system (Applied Biosystems, Darmstadt, Germany). Expression levels were normalized to β-actin. Reactions were done in duplicate using Applied Biosystems Taqman Gene Expression Assays and Universal PCR Master Mix. The relative expression was calculated by the 2 (-DDCt) method. The primers are shown as follows:

GLS: Forward TTCGCCCTCGGAGATCCTAC
Reverse CCAAGCTAGGTAACAGACCCT
GS: Forward CTGAGTGGAACTTTGATGGCT
Reverse GGAAGGGGTCTCGAAACATGG
SLC1A5: Forward CATCAACGACTCTGTTGTAGACC
Reverse CTGGATACAGGATTGCGGTATTT
SLC7A5: Forward CTACGCCTACATGCTGGAGG
Reverse GAGGGCCGAATGATGAGCAG

### Western Blotting Assays

Cell extracts were prepared according to the instruction of RIPA buffer (Biotek Corporation, Beijing, China). Cell lysates were collected by centrifugation at 12,000 rpm for 15min at 4°C, and then transferred to clean microcentrifuge tubes. Protein concentration was determined with Bradford reagent (Bio-Rad), and equal amounts of proteins (60 μg) were run on a 10 or 15% SDS–PAGE gel and blotted onto polyvinylidene fluoride membranes. After blocking for 1 h at room temperature with 5% non-fat dry milk, membranes were incubated with primary antibodies mouse anti-GS antibody (1:100, #ab64613, Abcam, USA), rabbit anti-H3k4me2 (1:1,000, #9725S, Cell Signaling Technology, USA), rabbit anti-LSD1 (1:1,000, #2139S, Cell Signaling Technology, USA), rabbit anti-p-mTOR (Ser2448) (1:1,000, #9964T, Cell Signaling Technology, USA), rabbit anti-mTOR (1:1,000, #9964T, Cell Signaling Technology, USA), rabbit anti-mTORC1 (1:1,000, #9964T, Cell Signaling Technology, USA), rabbit anti-tubulin (1:1,000, #2146S, Cell Signaling Technology, USA), rabbit anti-β-actin antibody (1:1,000, #8457S, Cell Signaling Technology, USA), or rabbit anti-LaminB (1:1,000, #12255S, Cell Signaling technology, USA) at 4°C overnight, respectively. After rinsed with TBST, the membranes were incubated with HRP-conjugated secondary antibodies goat anti-rabbit (1:2,000, #ZB-2301, ZSGB, China) and goat anti-mouse (1:2,000, #ZB-2305, ZSGB, China). The signals were stimulated with Enhanced Chemiluminescence Substrate (#NEL105001 EA, PerkinElmer) for 1 min and captured with a Bio-Rad ChemiDoc MP System (170–8280).

### Hematoxylin and Eosin Staining

Adipose tissue, kidneys, spleens, and livers were collected from mice. These tissues were fixed in 10% buffered formalin for 48 h and immersed in 30% sucrose for 48 h. The tissues were then cut into sections at an interval of 30 μm. The sections were then deparaffinized, hydrated, washed, and stained with hematoxylin and eosin (H&E) staining. Images were taken under a light microscope (Leica, Germany).

### Calculation of Inhibition Rates by 5FU *In Vitro* and *In Vivo*

**Figure 1:**
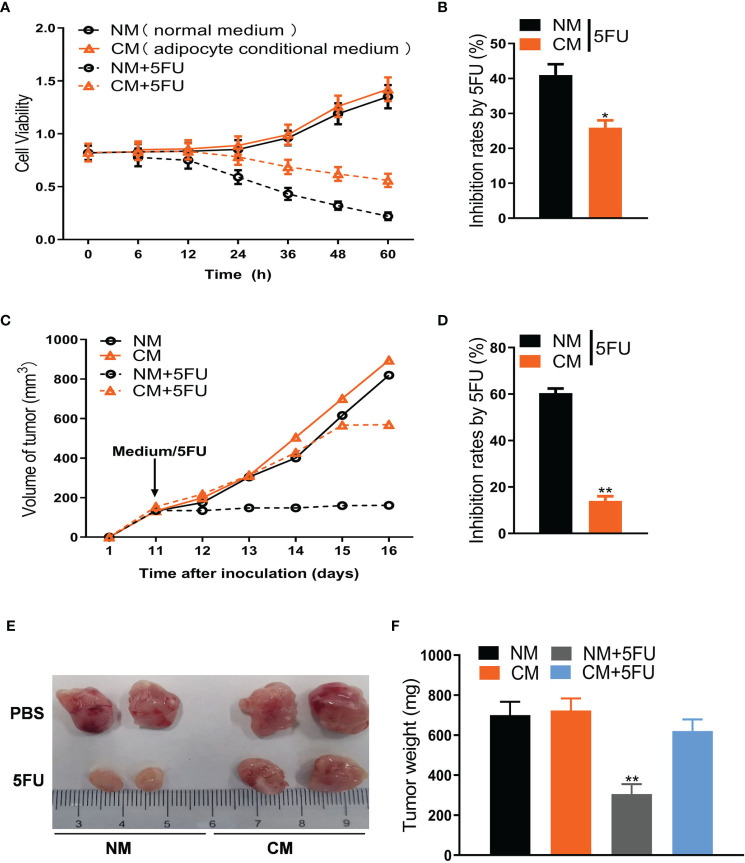
Adipose cells resist 5FU efficacy in CRC treatment. **(A)** Cell viability of CT26 cells treated with conditional medium and 5FU (10 μmol/L) by CCK8 assays (n = 5). **(B)** The inhibition rates of 5FU were measured according to **(A)** (n = 5, *P < 0.05). **(C)** Adipose cells induce resistance of CT26-tumors to 5FU treatment. CT26 cells (1.0 × 10^6^) were subcutaneously implanted in BALB/c mice. The treatment with 5FU and/or the conditional medium including NM, CM, and CM (5FU) was initiated when the tumor size reached to around 0.5 cm in diameter. The tumor size was measured dynamically. **(D)** The inhibition rates based on **(C)** were calculated (n = 5, **P < 0.01). **(E)** The representative images of CT26 tumors (on day 17) from the mice as described in **(C)**. **(F)** Weight of CT26 tumors (on day 17) from the mice as described in **(C)** (n = 5, **P < 0.01).

The cell viability was measured according to CCK8 assays. The cell growth curve of each group was displayed in the observed time period. The inhibition rate by 5FU was obtained by calculating the percentage of 5FU-reduced area under the curve in the observed time period. For example, the inhibition rate of NM group in **Figure 1B** was obtained by calculating the reduced-percentage of area (between the NM curve and NM+5FU curve) relative to the area under the NM curve in the observed time period in **Figure 1A**. *In vivo*, the tumor growth curve of each group was recorded. Likewise, the inhibition rate by 5FU was calculated as describe above.

### Statistical Analysis

For *in vitro* and *in vivo* results and metabolome data, statistical analysis was analyzed using SPSS 17.0 software (Version 17.0, LEAD Technologies, Chicago, USA). These data were expressed as mean ± SEM. Two-tailed unpaired Student’s t-test was utilized to analyze data between two groups. One-way ANOVA was used to analyze data among three groups with Turkey’s *post hoc* analysis. *P < 0.05. **P < 0.01. ***P < 0.001.

## Results

### Adipocytes Resist 5FU Efficacy in CRC Treatment

To explore the mechanism linking adipocytes to 5FU efficiency, we first collected the conditional medium (CM) of adipocytes differentiated from 3T3-L1 cells to treat the mouse CRC cells (CT26 cells) undergoing 5FU treatment. We showed that CM, but not normal medium (NM), notably weakened 5FU-induced proliferation inhibition of CT26 cells (**Figures 1A, B**). In mouse xenograft models, the inhibitory effect of 5FU on CT26-tumor size was largely blocked by CM (**Figures 1C, D**). Tumor weight reduced by 5FU was also rescued by CM (**Figures 1E, F**). Moreover, the effect of CM on 5FU resistance was confirmed by cell viability in human CRC cells like HCT-116 (**Figures S1A, B**) and SW-480 (**Figures S1C, D**). Cell cycle arrest assay showed that CM or 5FU did not affect cell cycle of CT26 cells. Whereas, Annexin V apoptosis assay showed that CM reversed the stimulatory effect of 5FU on apoptosis (**Figures S1E, F**). These results suggest that adipocytes induce chemoresistance to 5FU treatment for CRC.

### Adipocyte-Derived Glutamine Promotes Resistance to 5FU Chemotherapy

**Figure 2:**
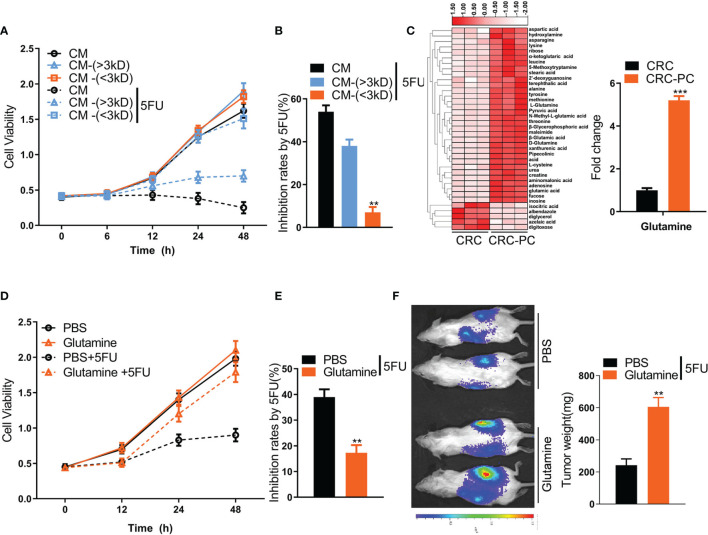
Adipocyte-derived glutamine (Gln) promotes resistance to 5FU chemotherapy in mouse CRC cells. **(A)** CM was separated into two fractions based on size: CM-(<3 KD) and CM-(>3 KD). The cell viability of CT26 cells treated with CM, CM-(<3 KD) or CM-(>3 KD) plus with/without 5FU (10 μmol/L) was measured. **(B)** The growth inhibition rates by 5FU were calculated according to **(A)** (n = 5, **P < 0.01). **(C)** Left, heatmap showing differential metabolic profiles of fat tissues from the peritoneum of the *in situ* CRC mode (CRC) (n = 3) and the peritoneal metastasis model (CRC-PC) (n = 3). The color represents the metabolite concentration of each sample calculated by peak area normalization method. Right, fold change of Gln level between the CRC-PC and the CRC group (n = 3, ***P < 0.001). **(D)** Cell viability of CT26 cells treated with 5FU (10 μmol/L), Gln (2 mM) or Gln (2 mM) plus 5FU (10 μmol/L). **(E)** Inhibition rates by 5FU according to the data in **(D)** (n = 5, **P < 0.01). **(F)** Gln-induced chemoresistance to 5FU. Left, tumor growth in peritoneal xenografts. Right, tumor weight. The tumors were isolated and weighed on day 17 (n = 5, **P < 0.01).

Dysfunctional adipocytes can secrete cytokines and metabolites, promoting proliferation, progression, and migration of cancer cells (29). To explore which factors from adipocytes are involved in chemoresistance to 5FU, we separated the CM of adipocytes into two fractions: cytokine fraction (>3 kD) and metabolite fraction (<3 kD). The inhibition rate by 5FU in CT26 cells was significantly reduced by metabolite fraction (<3 kD) rather than cytokine fraction (>3 kD) (**Figures 2A, B**). Then, we performed metabolomics analysis of fat tissues from the peritoneum of the peritoneal metastasis model and the *in situ* CRC model. We found that the Gln level was markedly increased in fat tissues in the CRC-PC model compared with the *in situ* CRC model (**Figure 2C**), indicating that Gln might induce chemoresistance to 5FU therapy, leading to PC. To verify this hypothesis, we tested the cell viability of CT26 cells treated with Gln or PBS. The data showed that Gln resisted 5FU-induced cell growth inhibition in CT26 cells (**Figures 2D, E**). In addition, the tumor weight was increased in Gln-treated intraperitoneal xenografts compared with PBS-treated intraperitoneal xenografts under the treatment of 5FU (**Figure 2F**), demonstrating the desensitizing effect of Gln on 5FU therapy. Moreover, we also verified that Gln induced resistance to 5FU chemotherapy in human CRC cells SW-480 (**Figures S2A, B**) and HCT-116 (**Figures S2C, D**). In mouse subcutaneous xenograft models, the 5FU-reduced tumor weight was rescued by Gln, while Gln alone did not affect tumor weight (**Figures S2E, F**).

### Adipocytic Gln Synthetase Promotes Chemoresistance to 5FU Therapy in CRC-PC

**Figure 3:**
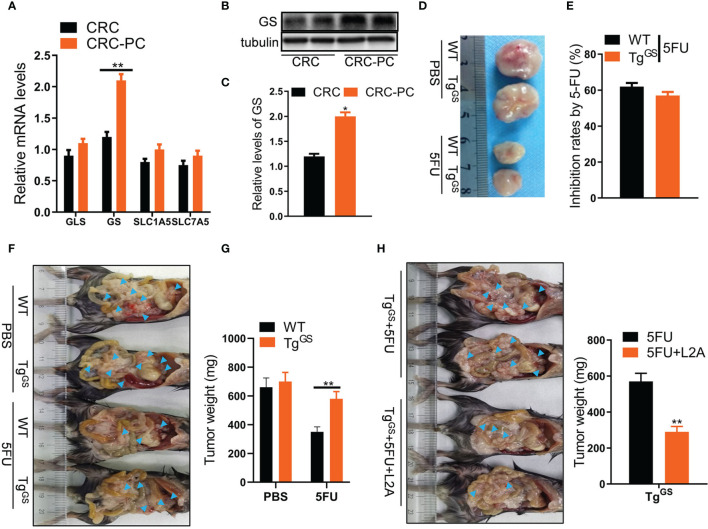
Adipocytic glutamine synthetase (GS) promotes chemoresistance to 5FU therapy in CRC-PC. **(A)** qPCR detecting relative mRNA levels of GLS, GS, SLC1A5, SLC7A5 in fat tissues from the peritoneum of the CRC model (n = 3) and CRC-PC (n = 3) **P < 0.01. **(B)** The protein levels of GS in the CRC-PC model and the CRC model were measured with Western blot. **(C)** The relative protein levels of GS in **(B)** were calculated (n = 3, *P < 0.05). **(D)** The representative images of MC38 tumors (on day 17) from the PBS-treated WT and GS transgenic (Tg^GS^) mice, 5FU-treated WT and Tg^GS^ mice. **(E)** The growth inhibition rates by 5FU in WT and Tg^GS^ mice (n = 5). **(F)** WT mice and Tg^GS^ mice were intraperitoneally inoculated with MC38 cells, and treated with PBS or 5FU for 2 weeks, respectively. Then, the representative images of the mice-bearing tumors were showed. Arrow heads, tumors. **(G)** The tumors in **(F)** were isolated and weighed (n = 3, **P < 0.01). **(H)** Left, Tg^GS^ mice were intraperitoneally inoculated with MC38 cells, and treated with 5FU or 5FU+L2A, respectively. Arrow heads, tumors. Right, the tumors were isolated and weighed (n = 3, **P < 0.01).

Given that Gln level can be regulated by enzymes GS and glutaminase (GLS) and amino acid transporters SLC1A5 and SLC7A5 (30), and that cancers cause dysregulation of GS and GLS (16), we detected relative mRNA levels of GLS, GS, SLC1A5, and SLC7A5 in fat tissues of mice with CRC or those of mice with CRC-PC by qPCR to evaluate the changes of Gln metabolism in CRC-PC. We found that GS expression was higher in the fat tissue from the peritoneum of the CRC-PC model compared with the *in situ* CRC model, while the expressions of GLS, SLC1A5, and SLC7A5 did not differ between the two groups (**Figure 3A**). We also detected the relative protein levels of GS by Western blot. In accordance with the mRNA level, the relative protein level was increased in the fat tissue from the peritoneum of the CRC-PC model compared with the *in situ* CRC model (**Figures 3B, C**). Next, we generated adipocyte-specific GS-expressing transgenic mice (Tg^GS^) and verified increased protein and mRNA levels of GS in Tg^GS^ mice compared with WT mice (**Figures S3A, B**). We also verified an increase in adipose-specific GS mRNA levels (**Figure S3C**). H&E staining of adipose, kidney, spleen, and liver showed no apparent difference between WT and Tg^GS^ mice (**Figure S3D**). Next, we established xenograft models in Tg^GS^ mice. The inhibition rates by 5FU showed no difference between 5FU-treated WT mice and 5FU-treated Tg^GS^ mice (**Figures 3D, E**). WT mice and Tg^GS^ mice were intraperitoneally inoculated with MC38 cells and treated with PBS or 5FU for 2 weeks. The tumor weight was reduced in 5FU-treated WT mice compared with non-treated WT mice, whereas 5FU-treated Tg^GS^ mice showed no difference with non-treated Tg^GS^ mice in tumor weight (**Figures 3F, G**). L2A, an antagonist of GS, was given to 5FU-treated Tg^GS^ mice. We found that the tumor growth was reduced in L2A+5FU-treated Tg^GS^ mice compared with 5FU-treated Tg^GS^ mice (**Figure 3H**). Further, GS was silenced by siRNA in adipocytes differentiated from 3T3-L1 cells, which was verified by the decreased GS mRNA level in si-GS-treated cells compared with si-NC-treated mice (**Figure S4A**). As expected, si-GS also reduced the relative glutamine level compared with si-NC (**Figure S4B**). In addition, CM from si-GS-treated adipocytes significantly increased 5FU-induced growth inhibition of CT26 cells compared with CM from si-NC-treated adipocytes (**Figure S4C**). The inhibition rates by 5FU were also reserved by CM from si-GS-treated adipocytes (**Figure S4D**). In mouse xenograft models, the resistance to 5FU on CT26 tumor weight was reversed by CM (si-GS) (**Figure S4E**). Those results indicate that adipocytic GS induces chemoresistance to 5FU treatment for CRC-PC.

### Loss of H3k4me2 Increases GS Expression in Adipose Cells

**Figure 4:**
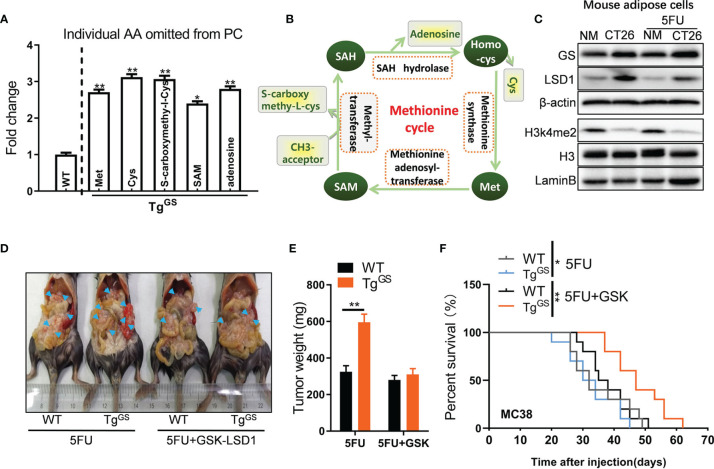
Loss of H3K4me2 increases GS expression in adipose cells. **(A)** Fold change of individual amino acid (AA) omitted from PC of WT and Tg^GS^ mice (n = 3, *P < 0.05, **P < 0.01). **(B)** Schematic diagram of methionine cycle. **(C)** The protein levels of GS, LSD1, H3K4me2, and H3 in non-treated or 5FU-treated mouse adipose cells cultured with NM or CT26 cell supernatants were measured with Western blot. **(D)** WT mice and Tg^GS^ mice were intraperitoneally inoculated with MC38 cells and treated with 5FU or 5FU+GSK-LSD1 for 2 weeks, respectively. Then, the representative images of the mic-bearing tumors were showed. Arrow heads, tumors. **(E)** The tumors in **(D)** were isolated and weighed (n = 5, **P < 0.01). **(F)** Kaplan-Meier curves for the survival of 5FU-treated or 5FU+GSK-LSD1-treated WT mice or TG^GS^ mice after MC38 tumor cell inoculation plotted against time (days after injection) (n = 10, **P < 0.01).

To detect metabolite changes induced by GS overexpression, we collected fat tissue samples from WT mice and Tg^GS^ mice and performed metabolomics. OPLS-DA score was calculated for each sample (**Figure S5A**), and differential metabolites of samples from WT mice and Tg^GS^ mice are shown in **Figure S5B**. The metabolomic data showed differentially expressed amino acid metabolites associated with the methionine cycle including methionine (Met), cysteine (Cys), S-carboxymethyl-L-cysteine, S-Adenosyl methionine (SAM), and adenosine (fold change > 2, *P*< 0.05) (**Figure 4A**). In the methionine cycle, Met is converted to SAM, the donor for epigenetic methylation, *via* methionine adenosyl-transferase. The methyl group is transferred to S-carboxymethyl-L-cysteine from SAM *via* methyltransferase (**Figure 4B**). SAM also provides methyl groups for DNA histone methylation (31). To explore the influence of CRC or 5FU treatment on histone methylation, we evaluated the expressions of several common histone methylation markers H3K4me2, H3K4me3, H3K9me2, H3K27me2, and H3K79me2 by Western blot in mouse adipose cells *in vitro*. The expression of H3k4me2, dimethylation at lysine 4 of histone H3, was reduced in mouse adipose cells cultured with CT26 cell supernatants compared with ones cultured with NM, while the expression of H3 did not differ between CT26 cell supernatant-treated adipocytes and NM-treated adipocytes (**Figure 4C**). LSD1 is a histone H3k4me2 demethylase, and we measured the expression of LSD1 in mouse adipocytes by Western blot. As expected, the expression of LSD1 was increased in adipocytes cultured with CT26 cell supernatants compared with ones cultured with NM. H3k27me2, dimethylation at lysine 27 of histone H3, showed reduced expression under 5FU treatment but no apparent difference in expressions when cultured with CT26 supernatants or NM (**Figure S5C**). The expressions of H3k4me3, H3k9me2, and H3k79me2 were affected by neither 5FU treatment nor CT26 supernatants. As expected, the expression of GS was increased in mouse adipose cells cultured with CT26 cell supernatants compared with adipose cells cultured with NM under the treatment of 5FU or non-treatment. GSK-LSD1 is a potent inhibitor of LSD1 and the inhibitory effect of GSK-LSD1 was confirmed by Western blot. Under the treatment of GSK-LSD1, the H3k4me2 expression was increased and the GS expression in adipocytes was reduced in a dose-dependent manner (**Figure S5D**). GSK-LSD1 also reduced the secretion of Gln in the supernatant of CT26 cells (**Figure S5E**). Furthermore, we demonstrated that the supernatant from MC38 suppressed expression of H3k4me2 in adipocytes, and this effect was rescued by GSK-LSD1 (**Figure S5F**). To evaluate the effect of H3k4me2 on GS-induced chemoresistance to 5FU, we administered GSK-LSD1 to tumor xenograft Tg^GS^ mice and treated the mice with 5FU. Under the treatment of 5FU, the tumor weight of tumor xenograft Tg^GS^ mice was increased compared with WT mice, indicating GS-induced resistance to 5FU treatment. After administrating GSK-LSD1, the effect of GS on drug resistance was blocked (**Figures 4D, E**). In addition, after MC38 tumor cell inoculation, Tg^GS^ mice with 5FU+GSK-LSD1 treatment showed better survival than Tg^GS^ mice with 5FU treatment (**Figure 4F**). Those results suggest that loss of H3k4me2 increases GS expression and GS-induced chemoresistance to 5FU therapy for CRC.

### Tumor Cells Outcompete Adipose Cells for Gln *via* mTORC1

**Figure 5:**
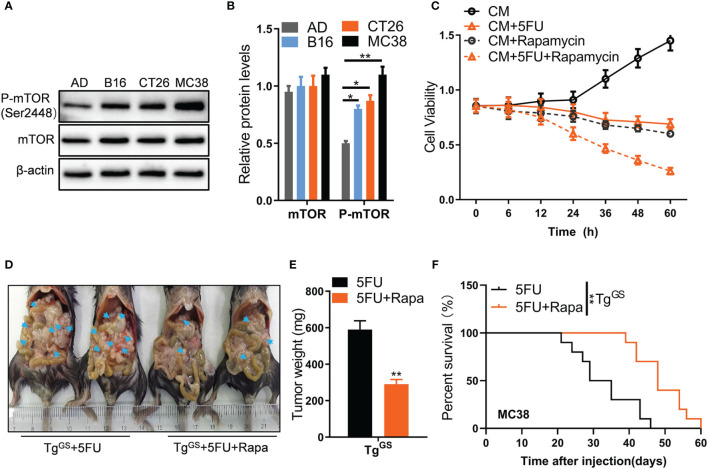
Tumor cells outcompete adipose cells for glutamine *via* mTORC1. **(A)** The protein levels of mTOR and p-mTOR (ser2448) in AD, B16, CT26, and MC38 cells were measured with Western blot. **(B)** The relative protein levels of mTOR and p-mTOR in **(B)** were calculated (n = 3, *P < 0.05, **P < 0.01). **(C)** Cell viability of CT26 cells treated with CM CM+5FU (10 μmol/L), CM+Rapamycin, or CM+5FU+Rapamycin by CCK8 assays (n = 5). **(D)** Mice were intraperitoneally inoculated with CT26 cells which were treated with 5FU, Rapamycin, or 5FU+Rapamycin, respectively. Then, the representative images of the mice-bearing tumors were shown. Arrow heads, tumors. **(E)** The tumors in **(D)** were isolated and weighed (n = 5, **P < 0.01). **(F)** Kaplan-Meier curves for the survival of 5FU-treated or 5FU+Rapamycin -treated TG^GS^ mice after MC38 tumor cell inoculation plotted against time (days after injection) (**P < 0.01).

Amino acid metabolism has been shown to participate in mTOR signaling (32, 33). To detect whether mTOR signaling is involved in adipocyte and GS-induced chemoresistance of CRC to 5FU, we established co-culture models of adipocytes and CRC tumor cells using mouse adipocytes differentiated from 3T3-L1 cells AD co-cultured with mouse tumor cell lines B16, CT26, and MC38, respectively. Then we utilized Western blot to measure relative protein levels of mTOR and p-mTOR. In the co-culture system, the relative protein level of p-mTOR (Ser2448) was higher in B16 cells, CT26 cells, and MC38 cells than in AD cells (**Figures 5A, B**), indicating that mTOR activation is increased in tumor cells. Furthermore, the expression of GS was increased in fat tissue of peritoneal in the CRC-PC model compared with that in the CRC model, while the expression of H3k4me2 was reduced in mice with CRC-PC compared mice with *in situ* CRC, p-mTOR no apparent difference in expressions (**Figure S6A**). To detect which of the two multi-protein complexes mTORC1 and mTORC2 has an effect on CRC cell survival, we added regulatory-associated protein of mTOR (Raptor) or rapamycin-insensitive companion of mTOR (Rictor), the component of mTORC1 and mTORC2, respectively (34), to CT26 cells. We found that Raptor, but not Rictor, inhibited survival rate of CT26 cells (**Figure S6B**). Then we knocked down mTORC1 by shRNA and verified it using Western blot (**Figure S6C**). The survival rate of CT26 cells was inhibited by mTORC1 shRNAs (**Figure S6D**). Moreover, tumor growth was also inhibited by mTORC1 shRNAs in mouse xenograft models (**Figures S6E, F**). Rapamycin, a selective mTOR inhibitor, was added to CM-treated CT26 cells for detecting whether mTOR was involved in chemoresistance to 5FU treatment. We found that the combined treatment of 5FU+Rapamycin had a stronger inhibitory effect on CT26 cell proliferation than 5FU treatment alone (**Figure 5C**), indicating that mTOR activation mediates resistance to 5FU therapy for CRC. Further, mouse tumor xenograft models were established in Tg^GS^ mice (**Figure 5D**). 5FU+Rypamycin reduced the tumor weight of xenograft Tg^GS^ mice compared with 5FU treatment alone (**Figure 5E**). In addition, after MC38 tumor cell inoculation, Tg^GS^ mice with 5FU+Rapamycin treatment showed better survival than Tg^GS^ mice with 5FU treatment (**Figure 5F**). These results suggest that mTOR activation mediated GS-induced chemoresistance to 5FU therapy for CRC.

## Discussion

**Figure 6:**
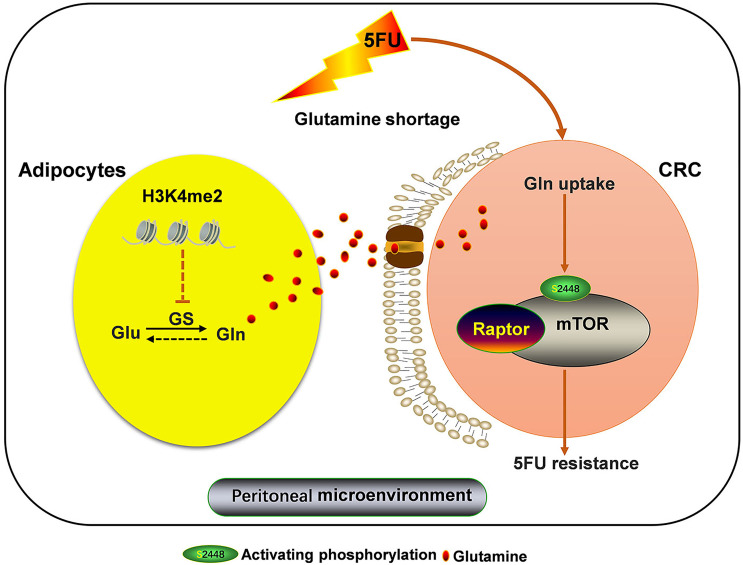
Adipocytic GS overexpression *via* altered histone methylation induces chemoresistance to 5FU in peritoneal carcinomatosis of colorectal cancer metastasis. Adipocyte-derived glutamine (Gln) promotes resistance to 5FU chemotherapy *via* inducing mTOR activation in CRC cells. Adipocyte-derived Gln production is increased by Gln synthetase (GS) upregulation, which is regulated by altered histone methylation. Loss of H3k4me2 increases GS expression in adipose cells.

Our present study reveals a novel mechanism of chemoresistance of CRC-PC to 5FU therapy. In the TME of CRC-PC, tumor cells outcompete adipocytes for Gln, leading to Gln deficiency. We show that this change in the TME induces GS upregulation in adipocytes, increasing the production of Gln, which promotes resistance of tumor cells to 5FU chemotherapy, a process mediated by mTOR activation. We also show that abnormal methionine metabolism in adipocytes may lead to altered H3k4me2 expression, which contributes to GS upregulation and chemoresistance to 5FU (**Figure 6**).

TME is a potential therapeutic target for metastasis and drug resistance (35, 36). TME consists of different cell types surrounding tumor cells, including fibroblasts, immune cells, endothelial cells, and adipocytes. The diversity of phenotypes in TME components may contribute to resistance to some therapies (36). Among TME components, adipocytes play an active role in chemoresistance. In ovarian cancer cells with adipocyte co-culture, adipocyte-induced demethylation reprograms cancer cell metabolism and promotes resistance to chemotherapy (37). *In vivo* studies have reported that adipocytes promote the metastasis of breast cancer (38, 39). In the current study, we displayed the pivotal role of adipocytes in promoting resistance to 5FU chemotherapy for CRC-PC. Some possible mechanisms underlying adipocytes-induced peritoneal metastasis include induction of angiogenesis *via* the CXCL2-VEGFA axis (40), activation of the PI3K/AKT/mTOR pathway by producing MCP-1 (41),upregulation of CD36 in tumor cells (42), and fatty acid metabolic reprogramming (43). Adipocytes also play key a role in drug resistance. The underlying mechanisms may include adipose hypoxia (44), alteration of pharmacokinetics (45, 46), variation of metabolic program (47, 48), production of tumor-promoting growth factors and cytokines (45), enhancement of cancer fibrosis (49), change of microbiota (50), generation of drug-resistant cancer stem cells (42), and cell-matrix adherence (51). Here we show a potential mechanism related to Gln metabolism.

Gln, the most abundant free amino acid in the human body, plays essential roles in various metabolic pathways and is involved in mitochondrial dysfunction and tumor cell proliferation (52). Gln metabolism is reprogrammed and plays an essential role in cancer (53). Alternations in amino acid metabolism in cancer result from increased nutritional demands of tumor cells for energy (54). In the present study, CRC cells outcompete adipocytes for Gln, thereby inducing adipocytes producing more Gln. We show that Gln is higher in fat tissue of patients with CRC-PC than in that of patients with CRC, suggesting the role of Gln in cancer metastasis. In human colon cancer cell lines, Gln promotes proliferation and inhibits differentiation, inducing a more aggressive phenotype (55). In the present study, we also demonstrated the desensitizing effect of Gln on 5FU treatment in mouse xenograft models. Drug resistance has been shown to be associated with amino acid metabolism (54), and Gln metabolism participates in resistance to chemotherapy in CRC (56).

GS is an ATP-dependent metalloenzyme that converts glutamate and ammonia to Gln. GS also contributes to endothelial cell motility and migration, promoting pathological angiogenesis (57). An increasing number of studies have revealed important roles of GS in cancers. The expression of GS was shown to be increased in liver, skeletal muscle, and kidney of rats implanted subcutaneously with fibrosarcoma (58, 59). GS overexpression was detected also in human primary liver cancers (60). Resistant hepatoma cell lines have more GS expression than sensitive cell lines (61). GS protein and mRNA levels were also increased in human breast cancer cell lines (62). Genetic deletion of macrophagic GS in tumor-bearing mice inhibited tumor metastasis (63). The current study shows that the protein level and mRNA level of GS are increased in fat tissue of patients with CRC-PC compared with that of patients with CRC. In addition, we displayed the stimulatory effects of GS on chemoresistance to 5FU therapy in mouse xenograft models. Further, our study revealed a mechanism of adipocytic GS-induced chemoresistance in CRC-PC *via* mTOR activation, in accordance with a precious study showing that Gln-dependent mTOR activation promotes chemoresistance in pancreatic cancer cells *in vitro* and *in vivo* (15). Amino acids can signal to mTORC1 and are the most crucial factors for mTORC1 activation (64, 65), but the mechanism remains largely unknown.

Alterations in histone methylation have a global influence on drug resistance in ovarian cancer cells (66, 67). In the present study, reduction of histone H3k4me2 methylation was observed in CRC cells and attributed to GS-induced chemoresistance to 5FU therapy, in accordance with a previous study which showed that H3k4me2 demethylation played an important role in CRC progression (68). In gastric cancer, LSD1 specifically catalyzed the demethylation of mono- and di-methylated H3k4me2, participating in many pathological processes of cancer, including proliferation, apoptosis, and metastasis (69). Overexpression of LSD1 has been proved in numerous cancers, and high level of LSD1 causes tumor aggressiveness and poor prognosis. Previous studies have also shown the synergistic antitumor effect of 5FU with the novel LSD1 inhibitor in colorectal cancer. However, whether LSD1 regulated by GS-Gln axis is far from well understood. The LSD1 inhibitor GSK-LSD1 exerted a great potential in translational medicine.

Taken together, the present study identifies an underlying mechanism of chemoresistance to 5FU therapy in CRC-PC *via* GS upregulation in adipocytes with subsequent release of Gln. Our findings demonstrate a crosstalk between histone methylation and GS metabolism in adipocytes and suggest that tumor methionine metabolism may be an efficient target for inhibiting adipocyte-induced chemoresistance in CRC-PC.

## Data Availability Statement

The raw data supporting the conclusions of this article will be made available by the authors, without undue reservation.

## Ethics Statement

The animal study was reviewed and approved by the Laboratory Animal Welfare and Ethics Committee of Army Medical University.

## Author Contributions

XZ, BW, and FP designed the research project. XZ performed mouse experiments and is a major contributor in writing the manuscript. QL performed Western blot and qPCR. AD performed cell cycle arrest assays and Annexin V apoptosis assays and proofread the revision manuscript. YL performed cell culture and cell assays. QS participated in manuscript writing. YC proofread the manuscript. YZ performed HE staining. BW and FP reviewed the manuscript. All authors contributed to the article and approved the submitted version.

## Funding

This work was supported by the National Natural Science Foundation of China (81702929) and Natural Science Foundation of Chongqing (cstc2020jcyj-msxmX0911).

## Conflict of Interest

The authors declare that the research was conducted in the absence of any commercial or financial relationships that could be construed as a potential conflict of interest.

## Publisher’s Note

All claims expressed in this article are solely those of the authors and do not necessarily represent those of their affiliated organizations, or those of the publisher, the editors and the reviewers. Any product that may be evaluated in this article, or claim that may be made by its manufacturer, is not guaranteed or endorsed by the publisher.
