# Supplementary material for: Adipocytic Glutamine Synthetase Upregulation via Altered Histone Methylation Promotes 5FU Chemoresistance in Peritoneal Carcinomatosis of Colorectal Cancer
Source: Front Oncol. 2021 Oct 12;11:748730. doi: 10.3389/fonc.2021.748730 (PMC8547656; doi:10.3389/fonc.2021.748730)
Supplement: Supplementary file 1 [file DataSheet_1.pdf]

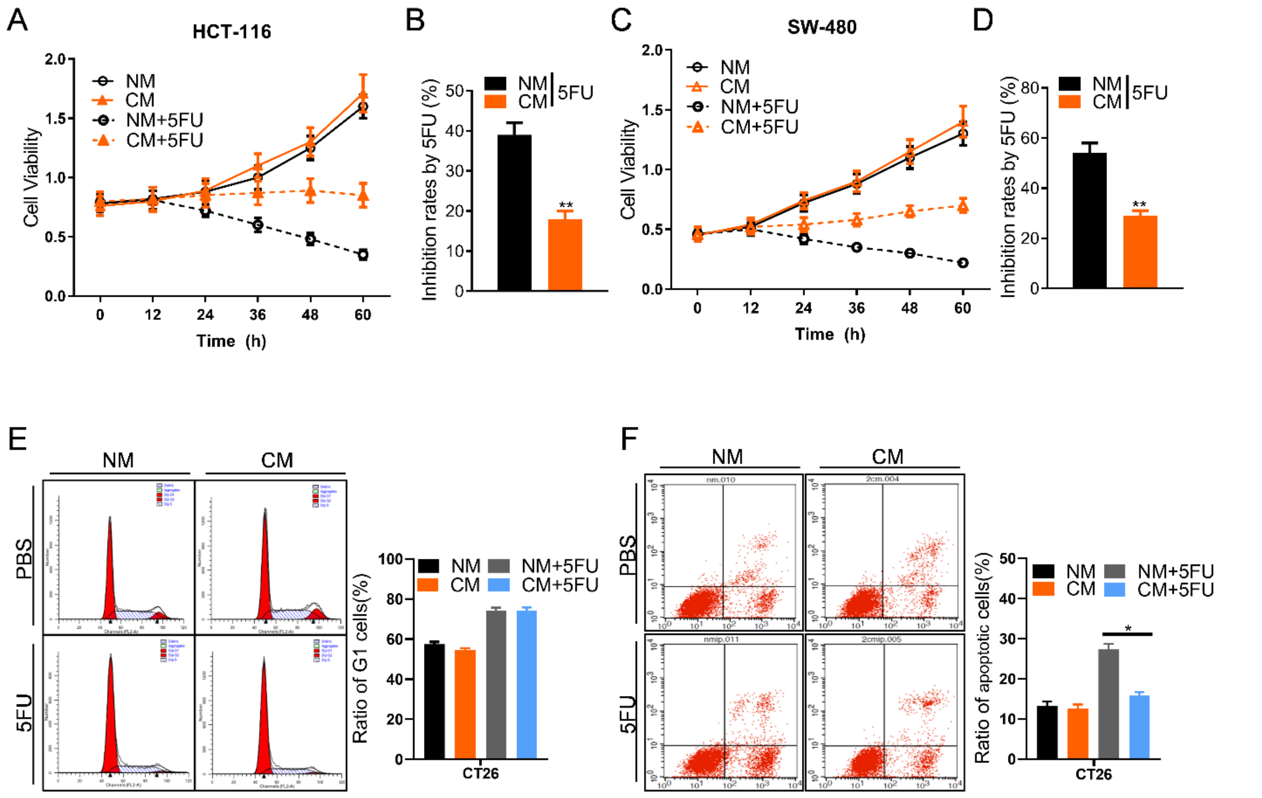

Supplementary Figure 1. Adipose cells resist 5FU treatment in human CRC cells. (A) Cell viability of HCT-116 cells treated with conditional medium and 5FU (10 μmol/l) by CCK8 assays (n = 5). (B) The inhibition rates of 5FU were measured according to (A) (n = 5, $^{**}P < 0.01$). (C) Cell viability of SW-480 cells treated with conditional medium and 5FU (10 μmol/l) by CCK8 assays (n = 5). (D) The inhibition rates of 5FU were measured according to (C) (n = 5, $^{**}P < 0.01$). (E) The cell cycle of CT26 cells treated with conditional medium and 5FU (10 μmol/l) by flow cytometry (n = 3). (F) The cell apoptosis of CT26 cells treated with conditional medium and 5FU (10 μmol/l) by flow cytometry (n = 3, $^{*}P < 0.05$).

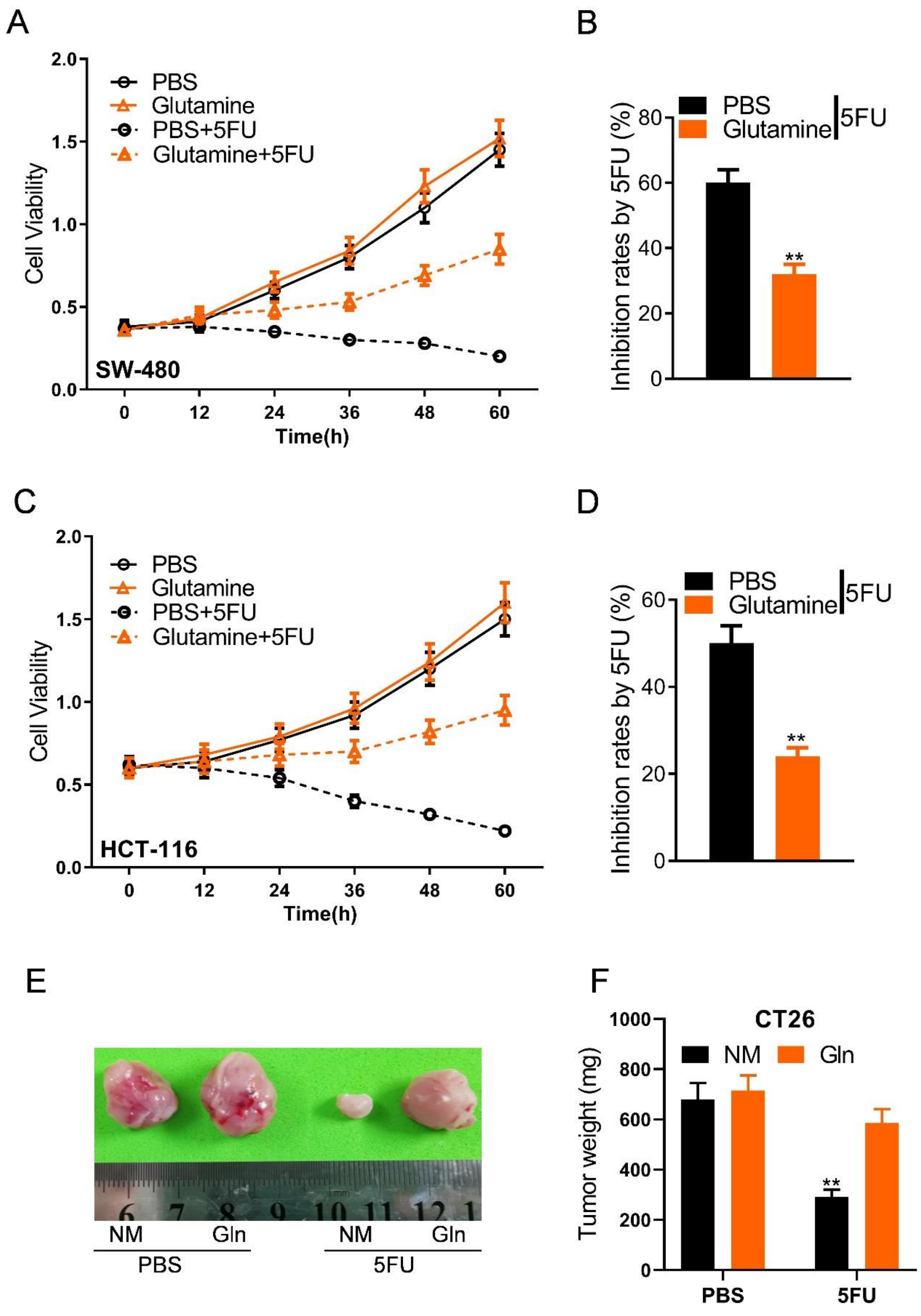

Supplementary Figure 2. Adipocytes-derived Gln promotes resistance to 5-FU chemotherapy in human CRC cells. (A) Cell viability of SW-480 cells treated with 5FU (10 μmol/l), glutamine (2 mM) or glutamine (2 mM) plus 5FU (10 μmol/l). (B) Inhibition rates by 5FU according to the data in (A) (n = 5, ***P* < 0.01). (C) Cell viability of HCT-116 cells treated with 5FU (10 μmol/l), glutamine (2 mM) or glutamine (2 mM) plus 5FU (10 μmol/l). (D) Inhibition rates by 5FU according to the data in (C) (n = 5, ***P* < 0.01). (E) The BALB/c mice were inoculated with CT26 tumors on day 1 and treated with Gln from day 5 and 5-FU (50 mg/kg.d) from day 11

Representative images of inoculated CT26-tumors (on day 17) were shown. (G) Tumor weight of the inoculated CT26-tumors was measured on day 17(n = 5, $^{**}P < 0.01$).

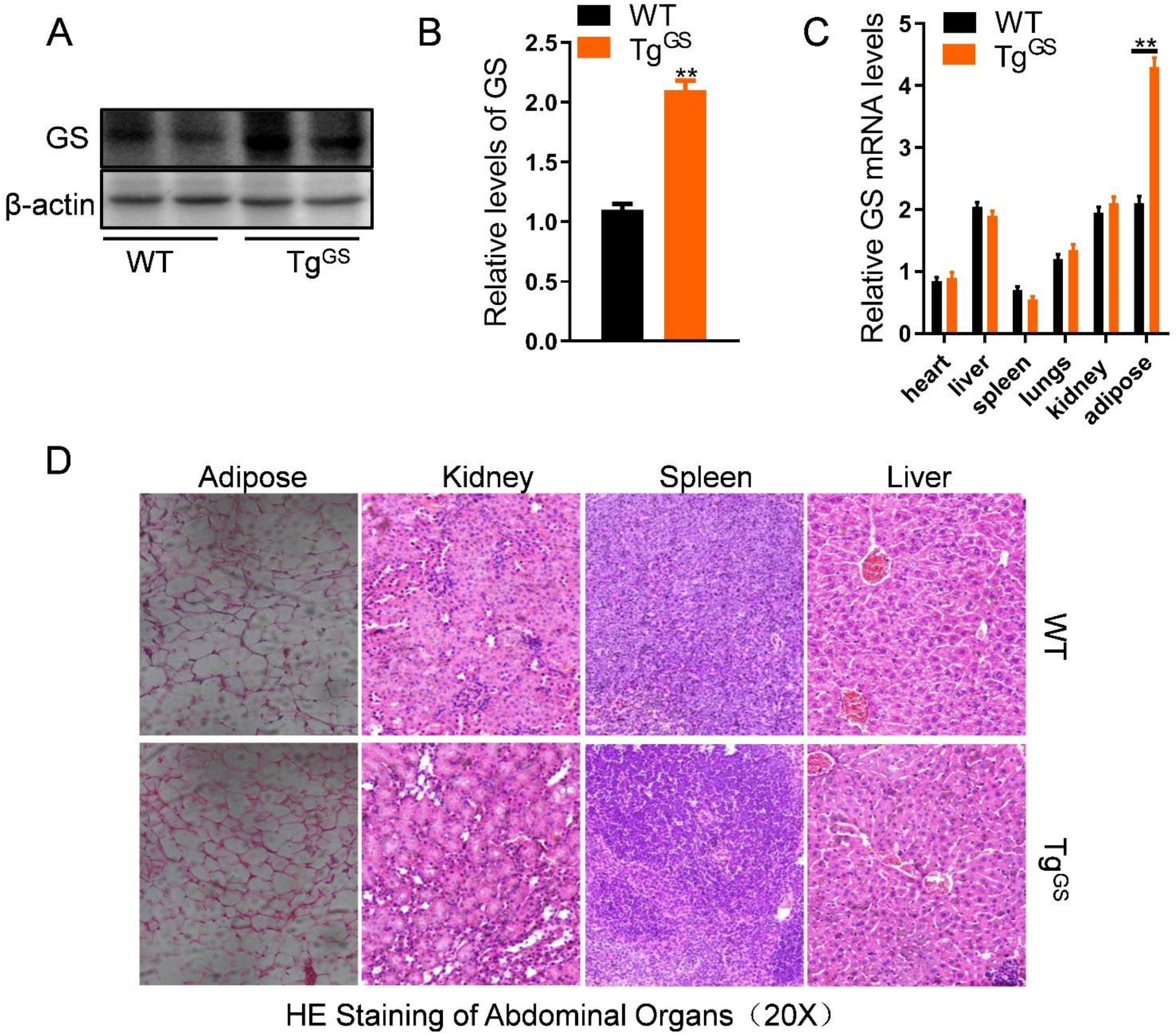

Supplementary Figure 3. Verification of Tg^{GS} mice. (A) Adipocyte- specific GS-expressing transgenic mice (Tg^{GS}) were generated. Representative Western blot images of GS in WT mice and Tg^{GS} mice were shown. (B) Quantification of relative protein levels of GS according to the data in (A) (n = 3, ***P* < 0.01). (C) qPCR detecting relative GS mRNA levels in various tissues (n = 3, ***P* < 0.01). (D) Representative H&E staining of various tissues of WT mice and Tg^{GS} mice.

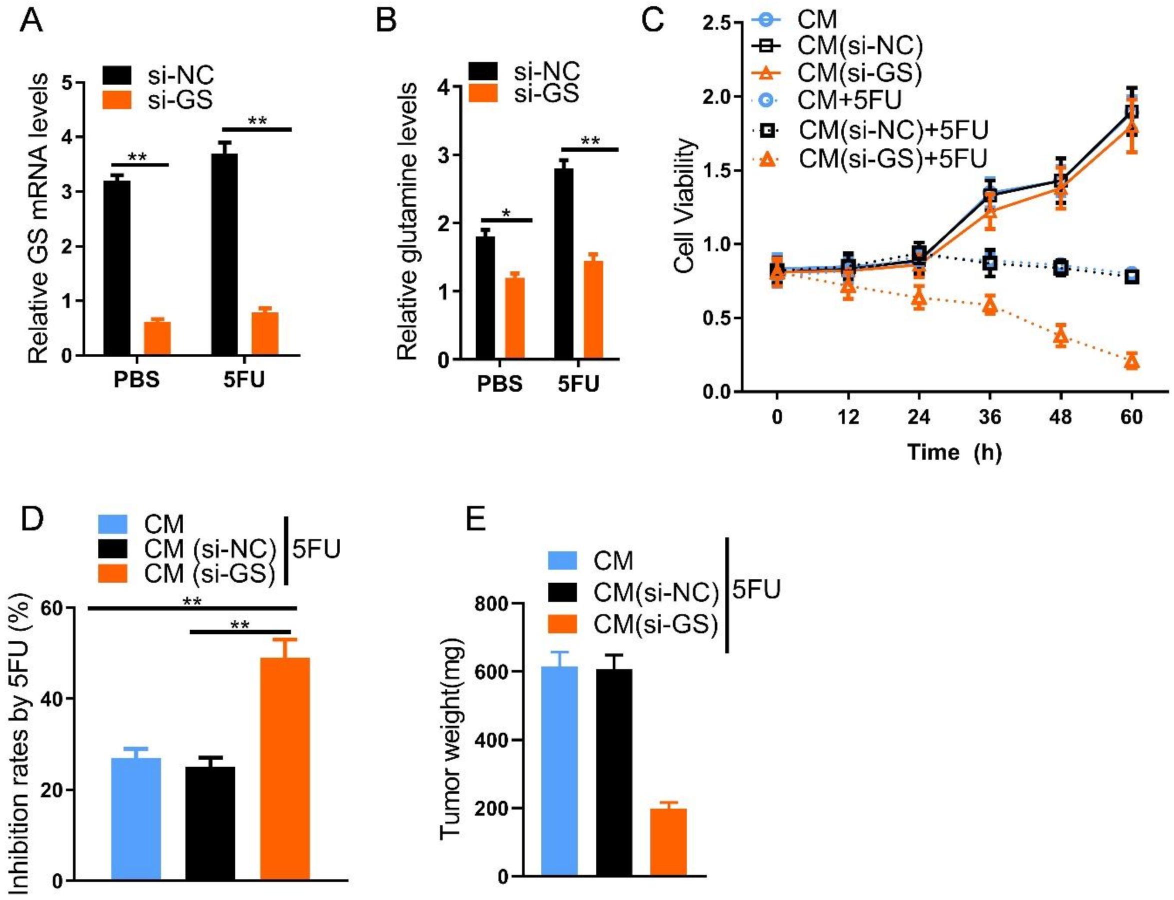

Supplementary Figure 4. GS knockdown in adipocytes sensitizes 5FU therapy in CRC. (A) Adipocytes differentiated from 3T3-L1 cells were treated with control siRNA (si-NC, 20 nmol/ml) or specific siRNA against mouse GS (si-GS, 20 nmol/ml) plus with/without 5-FU (10 μmol/l) for 24 h. Then, the relative mRNA levels of GS were determined by realtime PCR (n = 3, ***P* < 0.01). (B) Relative Gln levels in the supernatant of the adipocytes as described in (A) (n = 3, **P* < 0.05, ***P*< 0.01). (C) Cell viability of the CT26 cells treated with CM, CM (si-NC) or CM (si-GS) plus with/without 5FU (10 μmol/l). (D) The growth inhibition rates by 5FU were calculated from (C) (n = 5, ***P* < 0.01). (E) The BALB/c mice were subcutaneously inoculated with CT26 cells, and treated with CM (si-NC) or CM (si-GS) plus with/without 5FU for two weeks. Then, the representative weight of CT26 tumors (on day 17) were measured (n = 3, ***P* < 0.01)

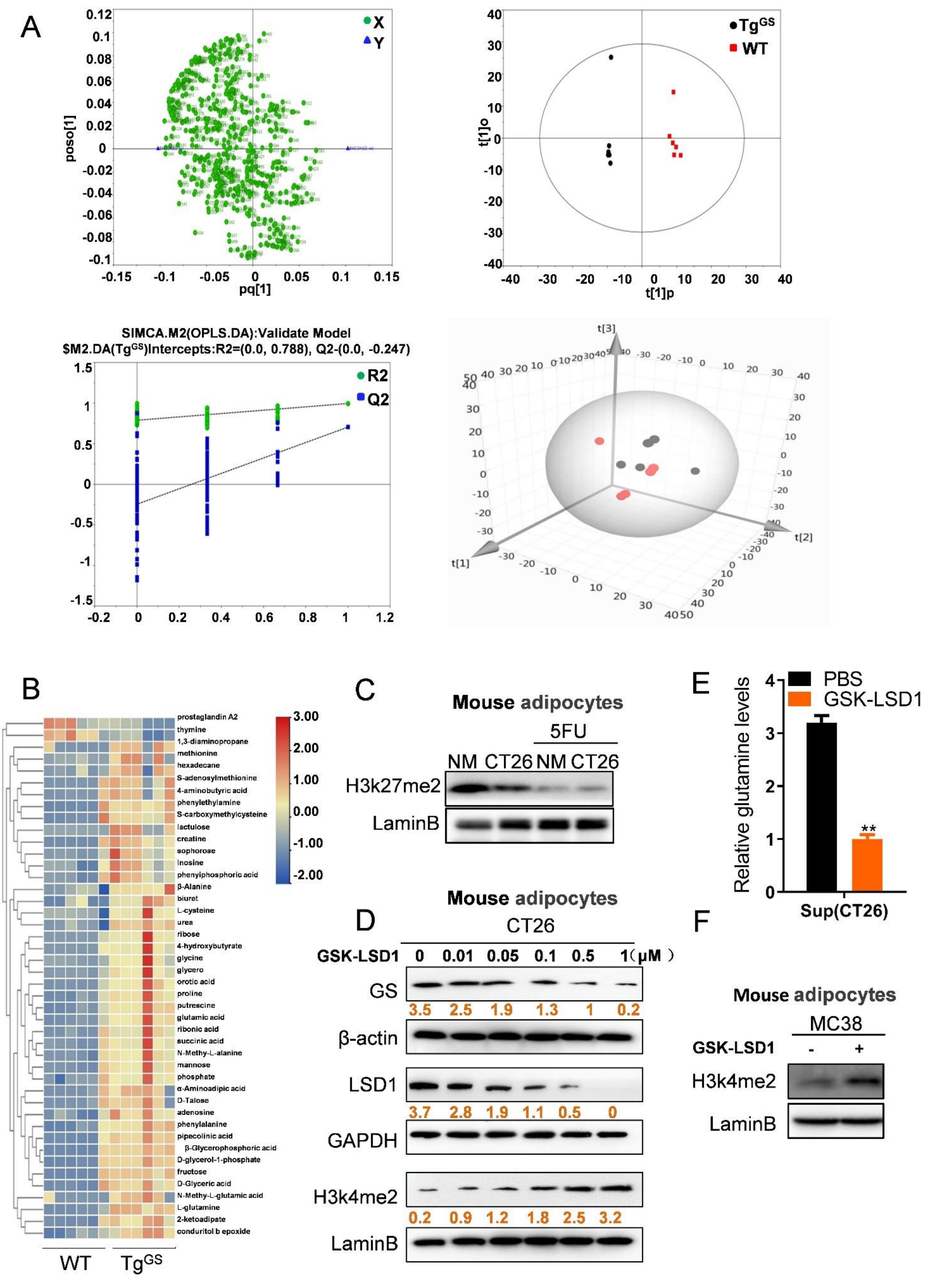

Supplementary Figure 5. (A) Principal component analysis (PCA) and Orthogonal projections to latent structures - discriminant analysis (OPLS-DA) of metabolomics data from fat tissues of WT mice (n = 6) and Tg^{GS} mice (n = 6). (B) Heatmap of differential metabolites from fat tissues of WT mice and Tg^{GS} mice. The color represents the metabolite concentration of each sample calculated by peak area normalization method. (C) Representative Western blot images of H3k27me2 in mouse adipose cells cultured with CT26 cell supernatants or NM. (D) Representative Western blot

images of GS, LSD1 and H3k4me2 in mouse adipose cells cultured with CT26 cell supernatants treated with GSK- LSD1 in different concentrations. Orange numbers, relative protein levels. (E) Relative Gln levels in the supernatant of the adipocytes as described in (D) (n = 3, $^{**}P < 0.01$). (F) Representative Western blot images of H3k4me2 in mouse adipose cells cultured with M38 cell supernatants treated with GSK- LSD1 or PBS.

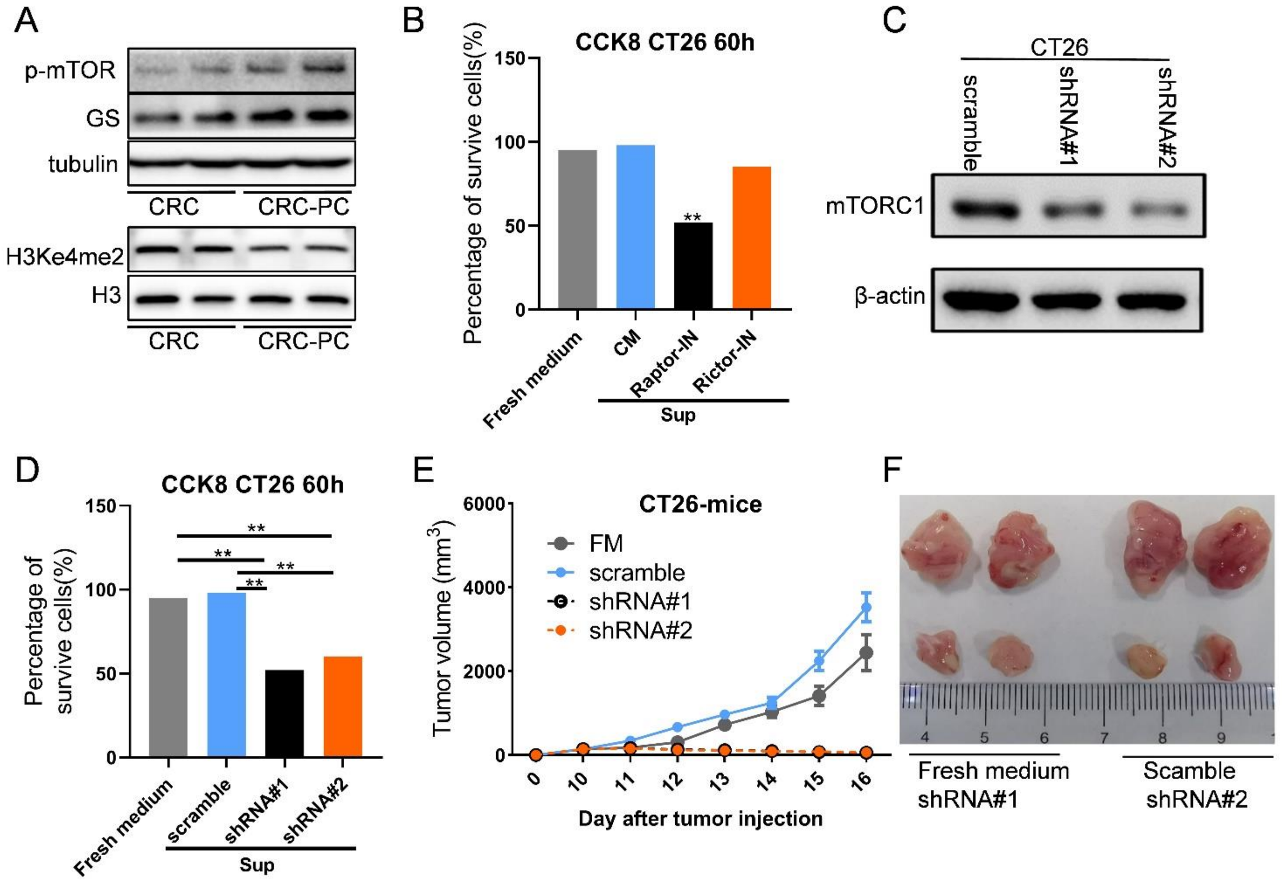

Supplementary Figure 6. (A) Representative Western blot images of p-mTOR, GS and H3k4me2 in fat tissues from the peritoneum of CRC or CRC-PC. (B) Raptor or Rictor, the component of mTORC1 and mTORC2 respectively, were added to CT26 cells. The percentage of survival rate (%) was measured in CT26 cells cultured in fresh medium or supplemented with CM, Raptor, or Rictor (n = 3, $^{**}P < 0.01$). (C) shRNA#1 and shRNA#2 were designed to knockdown mTORC1. Representative Western blot images of mTORC1 in CT26 cells transfected with scramble, shRNA#1, or shRNA#2. (D) The percentage of survival rate (%) was measured in CT26 cells cultured in fresh medium or supplemented with scramble, shRNA#1, or shRNA#2 (n = 3, $^{**}P < 0.01$). (E) CT26 cells (1.0×10^6) were subcutaneously implanted in BALB/c mice. The treatment with fresh medium, scramble, shRNA#1, or shRNA#2 was initiated when the tumor size reached to around 0.5 cm in diameter. The tumor size was measured dynamically. (F) The representative images of CT26 tumors (on day 17) from the mice as described in (E).
